# Supplementary material for: Does the Harvest Type Affect Olive Health? Influence of the Harvesting System and Storage Time on the Chemical, Volatile and Sensory Qualities of Extra Virgin Olive Oils
Source: Plants (Basel). 2023 Nov 14;12(22):3843. doi: 10.3390/plants12223843 (PMC10674536; doi:10.3390/plants12223843)
Supplement: Supplementary file 1 [file plants-12-03843-s001.zip › plants-2721163-supplementary.pdf]

Supplementary Material

Table S1. SM1- Volatile organic compounds detected from olive fruit and olive oil respectively.

Signals number	m/z	Chemical Formula	Tentative identification	Olive fruits	Olive oils
1	27.022	C2H3+	Acetylene	X	X
2	31.018	CH3O+	Formaldehyde	X	X
3	33.031	CH5O+	Methanol	X	X
4	41.038	C3H5+	Alkyl fragment	X	X
5	43.018	C2H3O+	Alkyl fragment (ethenone)	X	X
6	43.054	C3H7+	Hydrocarbon fragments	X	X
7	45.033	C2H5O+	Acetaldehyde	X	X
8	47.049	C2H7O+	Ethanol	X	X
9	51.038	CH7O2+	Water-methanol	X	-
10	53.038	C4H5+	Cyclobutadiene	X	X
11	55.054	C4H7+	Alkyl fragment	X	X
12	57.033	C3H5O+	2-Propenal	X	X
13	57.069	C4H9+	1-Butene (alkyl fragment)	X	-
14	59.049	C3H7O+	2-Propanone (acetone)	X	X
15	61.028	C2H5O2+	Acetic acid	X	X
16	65.038	C5H5+	Alkyl fragment	X	-
17	67.049	C5H7+	3-Penten-1-yne	X	X
18	69.069	C5H9+	2-Methyl-1,3-butadiene (isoprene)	X	X
19	71.049	C4H7O+	3-Buten-2-one	X	X
20	71.086	C5H11+	Alkyl fragment (several compounds)	X	X
21	73.065	C4H9O+	2-Butanone	X	X
22	75.044	C3H7O2+	Methyl acetate	X	X
23	77.038	C6H5+	Alkyl fragment	X	X
24	79.049	C6H7+	Benzene	X	X
25	81.069	C6H9+	C6 fragments	X	X
26	83.086	C6H11+	C6 fragments	X	X
27	87.080	C5H11O+	2-Methylbutanal	X	X
28	89.059	C4H9O2+	Ethyl acetate	-	X
29	91.040	C7H7+	Alkyl fragment (several compounds)	-	X
30	93.069	C7H9+	Terpene fragments	-	X
31	95.086	C6H7O+	C6 fragments	X	X

32	97.064	$C_6H_9O^+$	Dimethylfuran	X	X
33	99.080	$C_6H_{11}O^+$	Hexenal/methyl-pentenone	X	X
34	101.060	$C_5H_9O_2^+$	2,3-Pentanedione	X	X
35	105.069	$C_8H_9^+$	Styrene	-	X
36	107.086	$C_8H_{11}^+$	Ethyl benzene	-	X
37	115.075	$C_5H_{11}O_2^+$	Terpene fragments	X	X
38	121.101	$C_9H_{13}^+$	Terpene fragments	-	X
39	135.117	$C_{10}H_{15}^+$	p-Cymene	-	X
**Number of total VOCs detected**				32	36
